# Identifying Potential Lyme Disease Cases Using Self-Reported Worldwide Tweets: Deep Learning Modeling Approach Enhanced With Sentimental Words Through Emojis

**DOI:** 10.2196/47014

**Published:** 2023-10-16

**Authors:** Elda Kokoe Elolo Laison, Mohamed Hamza Ibrahim, Srikanth Boligarla, Jiaxin Li, Raja Mahadevan, Austen Ng, Venkataraman Muthuramalingam, Wee Yi Lee, Yijun Yin, Bouchra R Nasri

**Affiliations:** 1 Département de médecine sociale et préventive École de Santé Publique de l’Université de Montréal Université de Montréal Montréal, QC Canada; 2 Department of Mathematics Faculty of Science Zagazig University Zagazig Egypt; 3 Harvard Extension School Harvard University Cambridge, MA United States

**Keywords:** Lyme disease, Twitter, BERT, Bidirectional Encoder Representations from Transformers, emojis, machine learning, natural language processing

## Abstract

**Background:**

Lyme disease is among the most reported tick-borne diseases worldwide, making it a major ongoing public health concern. An effective Lyme disease case reporting system depends on timely diagnosis and reporting by health care professionals, and accurate laboratory testing and interpretation for clinical diagnosis validation. A lack of these can lead to delayed diagnosis and treatment, which can exacerbate the severity of Lyme disease symptoms. Therefore, there is a need to improve the monitoring of Lyme disease by using other data sources, such as web-based data.

**Objective:**

We analyzed global Twitter data to understand its potential and limitations as a tool for Lyme disease surveillance. We propose a transformer-based classification system to identify potential Lyme disease cases using self-reported tweets.

**Methods:**

Our initial sample included 20,000 tweets collected worldwide from a database of over 1.3 million Lyme disease tweets. After preprocessing and geolocating tweets, tweets in a subset of the initial sample were manually labeled as potential Lyme disease cases or non-Lyme disease cases using carefully selected keywords. Emojis were converted to sentiment words, which were then replaced in the tweets. This labeled tweet set was used for the training, validation, and performance testing of DistilBERT (distilled version of BERT [Bidirectional Encoder Representations from Transformers]), ALBERT (A Lite BERT), and BERTweet (BERT for English Tweets) classifiers.

**Results:**

The empirical results showed that BERTweet was the best classifier among all evaluated models (average F1-score of 89.3%, classification accuracy of 90.0%, and precision of 97.1%). However, for recall, term frequency-inverse document frequency and k-nearest neighbors performed better (93.2% and 82.6%, respectively). On using emojis to enrich the tweet embeddings, BERTweet had an increased recall (8% increase), DistilBERT had an increased F1-score of 93.8% (4% increase) and classification accuracy of 94.1% (4% increase), and ALBERT had an increased F1-score of 93.1% (5% increase) and classification accuracy of 93.9% (5% increase). The general awareness of Lyme disease was high in the United States, the United Kingdom, Australia, and Canada, with self-reported potential cases of Lyme disease from these countries accounting for around 50% (9939/20,000) of the collected English-language tweets, whereas Lyme disease–related tweets were rare in countries from Africa and Asia. The most reported Lyme disease–related symptoms in the data were rash, fatigue, fever, and arthritis, while symptoms, such as lymphadenopathy, palpitations, swollen lymph nodes, neck stiffness, and arrythmia, were uncommon, in accordance with Lyme disease symptom frequency.

**Conclusions:**

The study highlights the robustness of BERTweet and DistilBERT as classifiers for potential cases of Lyme disease from self-reported data. The results demonstrated that emojis are effective for enrichment, thereby improving the accuracy of tweet embeddings and the performance of classifiers. Specifically, emojis reflecting sadness, empathy, and encouragement can reduce false negatives.

## Introduction

Global warming and milder winters are causing the range of tick vectors to expand, which in turn is contributing to an increase in the incidence of tick-borne diseases [1-4]. Lyme disease is one of the most commonly reported tick-borne diseases worldwide [5]. In North America, Lyme disease is endemic in the northeastern, upper mid-West, and mid-Atlantic portions of the United States, and is prevalent in the southern regions of Canada [6-8]. In Europe, Lyme disease is mainly found in the central regions of the continent and in Scandinavian countries, and it is also found in Russia [6,9,10]. The occurrence of Lyme disease is very recent in Asia and has been reported in India, Turkey, China, Korea, Nepal, Taiwan, and Japan [11-16]. Owing to the current wide geographical spread of this disease, the early detection of potential Lyme disease cases will remain a public health concern in the forthcoming decades [17].

Lyme disease is caused by spirochetal bacteria that are part of the *Borrelia burgdorferi sensu lato* (*s.l.*) complex [17]. The *Borrelia burgdorferi sensu lato* (*s.l.*) complex contains numerous genospecies, but only a few can infect humans and cause Lyme disease. The genospecies that can infect humans have distinct geographic distributions: *Borrelia burgdorferi sensu stricto* is primarily found in North America, whereas *Borrelia afzelii* and *Borrelia garinii* are both prevalent in Asia and Europe [18,19]. Furthermore, the clinical manifestations of Lyme disease vary depending on the genospecies involved in the infection, and thus, the symptoms also vary by geographical region. In North America, *B. burgdorferi sensu stricto* (*s.s.*) typically causes Lyme arthritis and carditis, whereas both *B. afzelii* and *B. garinii* cause neuroborreliosis in Europe and Asia [9,20,21].

The infectious agent is transmitted to humans by several species of ticks from the *Ixodes* genus, whose distribution varies geographically. *Ixodes scapularis* and *Ixodes pacificus* are the most prevalent in North America, *Ixodes ricinus* is the most prevalent in Europe, and *Ixodes persulcatus* is the Lyme disease vector in Asia [16,18,22-24]. Tick vectors progress through sequential life stages: egg, larva, nymph, and adult. Ticks feed on hosts of different sizes throughout their growth stages. Specifically, nymphs primarily feed on rodents (especially *Peromyscus leucopus*, also known as white-footed mice), whereas adult ticks prefer larger mammals such as white-tailed deer (*Odocoileus virginianus*) [25].

Lyme disease has been called “the great imitator” in the literature because its clinical spectrum mimics various other unrelated diseases, making the correct diagnosis of Lyme disease based solely on clinical manifestations a difficult task, which can lead to misdiagnosis and mistreatment [26]. Lyme disease typically presents in 3 stages: early localized stage, early disseminated stage, and late disseminated stage [27,28]. The most common and usually first symptom of the early localized stage is a nonpruritic and painless rash with an erythematous center called erythema migrans (also known as the “bull’s-eye”) [27]. This symptom is present in nearly 90% of all Lyme disease cases and is accompanied by flu-like symptoms, including fever, headache, fatigue, adenopathy (lymph node), myalgia, and arthralgia [29]. The second stage is characterized by multiple skin and organ lesions, occurring months after exposure to the infected tick bite [26]. The heart, joints, and skin are the most affected organs [30-32]. The symptoms in the second stage include carditis (heart block, myocarditis, syncope, palpitations, dyspnea, and chest pain) and arthritis, which are most common in North America [31,33]. The late disseminated stage is manifested predominantly by neurological symptoms (radiculopathy, neck stiffness, meningitis, facial nerve palsy, cranial neuropathy, etc) [34,35]. Acrodermatitis chronica atrophicans and borrelial lymphocytoma are rare cutaneous manifestations of the third stage, and they are mostly noted in Europe and Asia [36].

The standard laboratory diagnosis of Lyme disease involves a 2-tier test in which an initial ELISA (enzyme-linked immunosorbent assay) screening test result is confirmed later by a western blot or an immunoblot [37]. Lyme disease is treatable with a short course of antibiotics, but if left untreated, it may lead to severe neurological, cardiac, and articular complications [38]. There is currently no vaccine against Lyme disease, and therefore, the only preventive measures are self-protection against tick bites and yard management [39].

Surveillance is one of the public health tasks aiming to monitor trends in disease epidemiology, identify populations at risk, and report disease cases [40,41]. Surveillance systems are based on active or passive surveillance approaches. Active surveillance is a surveillance system based on periodic collection of samples or case reports from health authorities, whereas passive surveillance is a system based on reporting of clinical suspect cases to the health authorities and depends on patient willingness to seek medical attention [40,42]. In North America (both the United States and Canada), Lyme disease reporting is compulsory, and the task falls on busy health care professionals to do so promptly [43,44]. In comparison, Lyme disease reporting is not mandatory in all endemic countries in Europe; however, the European Union recently called to standardize Lyme disease reporting and make it a notifiable disease [41]. Underreporting is a concern in Lyme disease epidemiology because the traditional surveillance system has failed to track all cases accurately [45-47]. For example, a recent study estimated the number of Lyme disease cases in the United States at over 400,000, while the Centers for Disease Control and Prevention (CDC) reported only 30,000 cases [45,48]. The United States is not the only country where underreporting of Lyme disease cases has been suggested, as this issue has been pointed out in some European countries as well [49-51].

According to a review conducted previously [26], the traditional Lyme disease surveillance system is prone to overreporting or underreporting due to multiple reasons. One reason is that the system is dependent on the reporting of cases by busy health care professionals, and therefore, only cases seen and diagnosed by professionals are reported. Another contributor to the deficiency of the Lyme disease surveillance system is the lack of accuracy of serologic tests for Lyme disease diagnosis. The clinical diagnosis of Lyme disease is based on clinical manifestations, appropriate serology, and a history of exposure to tick bites [35]. The interpretation of the results of serologic tests as positive indicators of Lyme disease is however problematic since these tests are not very sensitive in the early stage and can show false-negative results, thereby rendering treatment ineffective [52,53]. Therefore, some cases tend to get missed by health care professionals, especially in new areas, resulting in underreporting of the disease [37,48,54,55]. Additionally, the heterogeneity of Lyme disease bacterial strains contributes to the late diagnosis of Lyme disease cases [56,57]. Moreover, Lyme disease monitoring also depends on data collected from tick surveillance. Tick data can be collected through passive surveillance, which can provide insights about risk areas for tick-borne diseases, such as Lyme disease, and active tick surveillance can identify regions where tick populations are established [58,59]. In countries, such as China, where Lyme disease is not yet endemic and is not a notifiable disease, active tick surveillance is used to monitor Lyme disease cases and quantify infection risk [60].

With the extended use of the internet and social media platforms where health-related information is often shared, researchers have found an opportunity to improve disease surveillance systems by leveraging web data [61,62]. This new field of research is referred to as digital surveillance or infodemiology [63]. Among all current social media platforms, Twitter is one of the most popular social media platforms, with over 145 million daily active accounts, and it is the most widely used data source for digital health owing to certain advantages: its data can be easily accessed through the Twitter application programming interface (API), the size of the text (tweets) is limited to 280 characters (140 characters before 2017), and it is possible to geolocate the tweets [64]. A recent systematic review suggested that less than half of existing studies on digital health surveillance using Twitter data were focused mainly on prediction, and only a few studies focused on developing tools for adequate analysis of these types of data [61]. Owing to the novelty of digital surveillance in public health research, there is an unevenness in the different methodological approaches and data sets used [63]. Given the availability and the richness of text data from Twitter or from other platforms (such as Reddit), there is a need to develop reliable and accurate classification methods to process and analyze the data to study health-related issues [62]. Specifically, the development and evaluation of methodological machine learning approaches are often required for data analysis. In addition to a significant time investment, these activities usually require access to organized, validated, pretrained, and labeled data sets for various health problems to facilitate their development.

Several studies have used data from search engines and social media platforms to track Lyme disease [65-67]. For example, a previous study examined how the content of Lyme disease videos on YouTube differed depending on data sources and the people who produced the videos [65]. It was reported that public health experts did not produce popular videos on YouTube about Lyme disease. In addition, responsible reporting and innovative knowledge translation through videos can increase awareness of Lyme disease. To better forecast the incidence of Lyme disease in Germany, the authors in a previous study used digital data such as Google Trends [66]. While the official reported incidence of Lyme disease correlates well with Google Trends data, it did not significantly increase the forecasting accuracy. In another study, the prevalence of Lyme disease and the frequency with which the term “Lyme” was searched in Google Trends were examined in southern Ontario, Canada, between 2015 and 2019, resulting in the identification of a single hotspot in eastern Ontario [67]. Additionally, there was an increase in Google Trends for the term “Lyme disease,” which was associated with a significant increase in Lyme disease risk. According to previous studies, the number of Lyme disease searches in search engines was related to seasonal and geographic patterns of Lyme disease cases [68,69]. However, there are very few studies focusing on Lyme disease and social media. For example, a previous study showed that Twitter can be used to monitor Lyme disease through the use of Twitter data (tweets) as a proxy for monitoring disease prevalence in the United Kingdom and the Republic of Ireland [70]. A limited geographic search strategy was used to discover spatial patterns and find rare cases of Lyme disease. Another study reported that Lyme-relevant Twitter data are correlated with official reports on the disease in the United States [71].

Our study aims to fill the gap on the use of web data to study Lyme disease in a very useful way. Indeed, our study seeks to provide an accurate English worldwide tweet data set and evaluate the performance of some selected natural language processing (NLP) transformer-based models, including DistilBERT (distilled version of BERT [Bidirectional Encoder Representations from Transformers]), ALBERT (A Lite BERT), and BERTweet (BERT for English Tweets), by integrating emotional component emojis. We believe that the novelty and completeness of this data set will assist in the development and evaluation of digital Lyme disease surveillance systems and will be a useful resource for public health researchers and practitioners. It is important to mention that this study is a continuation of a recent study where a machine learning–based model has been proposed for predicting Lyme disease cases and incidence rates in the United States using Twitter [71]. However, unlike this previous study, our work here provides a worldwide data set of English tweets and evaluates the performance of the selected advanced machine learning transformer–based models with the integration of emojis, which will lead to new and more accurate classified data for Lyme-related tweets.

This study has the following objectives:

1. Provide an openly available data set to the scientific community for its use in a variety of experimental epidemiological research, at a time when there is an urgent need to integrate novel web-based data sets related to the Lyme disease epidemic (such as this classified data set) with other data sets from other sources for improving risk prediction.
2.  Analyze the performance of several prominent NLP classifiers in terms of their ability to predict potential cases of Lyme disease. 
3. Evaluate the effect of incorporating emojis as enrichment features to improve the performance of the transformer-based classifier.
4. Determine whether specific patterns could be identified regarding the prevalence of Lyme disease for each country based on the classified tweets.

## Methods

### Overview

**Figure 1:**
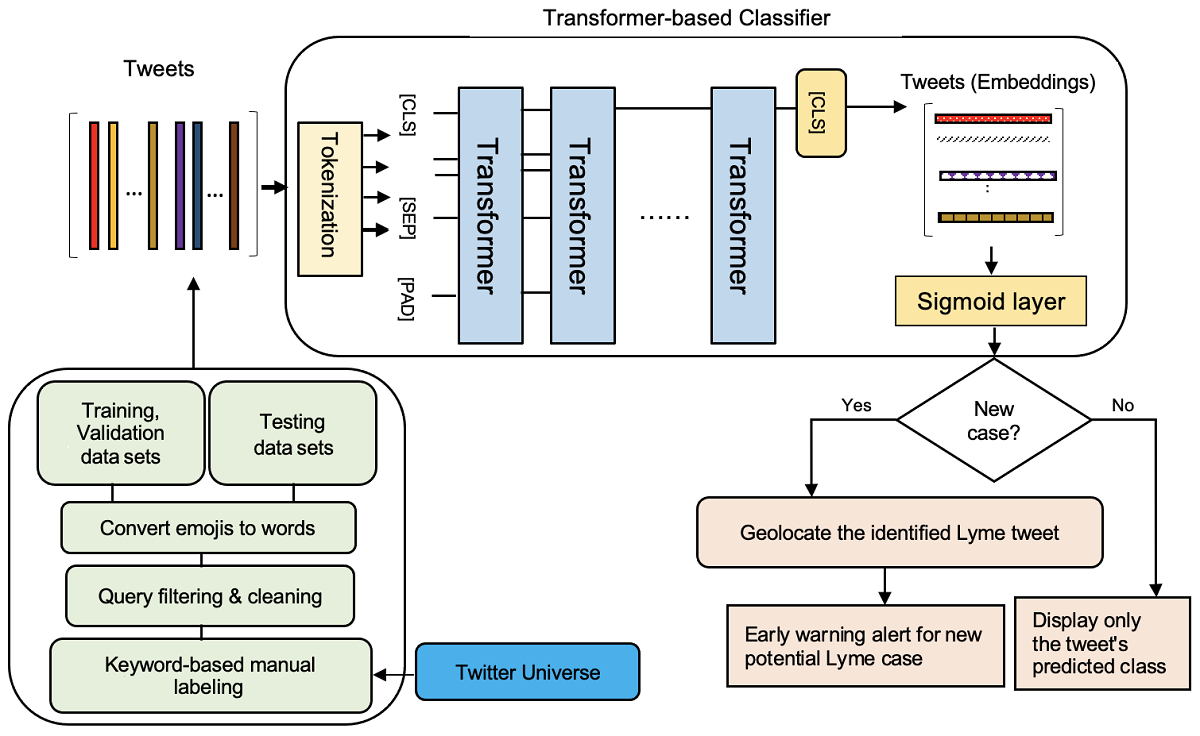
The 2-stage approach proposed for predicting potential Lyme disease cases. The first stage involves 4 elements: (1) We used standard search terms to collect tweets via the Twitter application programming interface; (2) We cleaned the tweets by removing hashtags, URL links, HTML markups, and stop-words; (3) We manually labeled the tweets as Lyme or non-Lyme using a list of precise keywords; and (4) We converted emojis into sentiment words, which were then substituted for the emojis in the tweets. In the second stage, we used a transformer-based classifier to determine whether a tweet is a potential Lyme disease case or not. When a new tweet was assigned with the highest probability to the Lyme disease class, we used the GeoPy library to estimate the tweet’s location. The 3 special tokens were as follows: [CLS], which stood for classification and was typically the first token of every sequence; [SEP], which described to the pretrained language model which token belongs to which sequence; and [PAD], which was used to fill the unused token slots to ensure that the maximum token length was met.

Owing to the nature of Twitter data, the analysis requires developing and evaluating machine learning–based methodologies [61,72]. Figure 1 illustrates the methodology used to classify the tweets. This process consists of the following 2 key elements: (1) collecting and preprocessing self-reported Lyme-related tweets and (2) identifying potential Lyme-disease cases.

### Data Collection and Preprocessing

Using an academic research account with Twitter’s API and search terms like “#Lyme” and “#Lyme disease,” about 20,000 English tweets were collected between 2010 and 2022. Tweets were cleaned to reduce text noise and redundancy by deleting hashtags, URL links, HTML markups, stop-words, username mentions, and retweets. Accurate keywords or search terms are required to properly label extracted information from social media. Keywords used to label the collected data are important as they will impact the results and the quality of surveillance. Many studies have attempted to improve the relevance of disease-related keywords by examining word frequency using a corpus of tweet text and labeling approaches [72,73]. As such, we compiled a list of precise keywords that are often associated with Lyme disease. These keywords were used as the basis of regular expression that was applied to the cleaned tweets to manually determine whether they were relevant to Lyme disease. A label “1” was assigned to potential Lyme disease–related tweets, while “0” was assigned to those that were not related. As mentioned in a previous report [71], we used 2 methods to specify keywords in regular expression. The first method entailed investigating the content of the cleaned tweets to determine the relative frequency of common colloquial Lyme disease words such as *have Lyme*, *had Lyme*, *having Lyme*, *has Lyme*, *get Lyme*, *gets Lyme*, *got Lyme*, *getting Lyme hiking*, *hike*, *forest*, *tick*, *ticks*, *bite*, *deer*, *deertick*, and *tickborne*. By using this method of keyword selection, Twitter posts like “She is terribly unwell, we suspect it's Lyme” were labeled as potential Lyme disease cases. The second method of keyword selection involved considering the most frequent Lyme disease symptoms, transmission channels, or scientific terms, such as *erythema migrans*, *carditis*, *fever*, *rash*, *headache*, *fatigue*, *chills*, *nausea*, *vomiting*, *dizziness*, *sleepiness*, *hallucinations*, *depression*, *numbness*, *tingling*, *facial paralysis*, *palpitations*, *borrelial lymphocytoma*, *anxiety*, *memory loss*, *joint aches*, *muscle aches*, *swollen lymph nodes*, *neck stiffness*, *nerve pain*, *arthritis*, *shortness of breath*, *irregular heartbeat*, *shooting pains*, *skin redness*, *tick bite*, and *acrodermatitis chronica atrophicans*.

Therefore, tweets, such as “I suffered from Lyme symptoms four years ago” and “My sister is developing fever after a tick bite,” were also labeled as potential Lyme disease cases. It is important to note that this manual approach aims to ensure fair and accurate labeling of the data set. This is because automatically labeling tweets with off-the-shelf Python regular expression libraries does not always provide correctly labeled tweets. It could also be argued that there are differences between the 2 keyword selection methods we used to manually label the tweets. However, the rationale here was based on the fact that the use of “Lyme disease” keywords in search engines has been demonstrated to improve results [68]. Therefore, the first method of keyword selection can be viewed as a naive general way to label any Lyme disease data collected from web-based sources, whereas the second method is a more specific and accurate way to confidently label tweets as related to Lyme disease. We would like to point out that, due to a lack of resources, each manually labeled tweet was only reviewed by 1 person. However, all reviewers agreed on a guideline, and several examples were provided to simplify understanding of the categories and reduce misclassification.

The labeled data set of 20,000 cleaned tweets was split into 3 disjoint data sets. Initially, a training data set (n=12,000) was compiled by randomly selecting exactly 1000 tweets from each year between 2010 and 2022. All tweets were distributed between 2 classes based on prior manual classification: potential Lyme disease cases (n=6000) and non-Lyme disease cases (n=6000). The remaining tweets (n=8000) were then separated into 2 equal parts: a validation data set (n=4000) and a testing data set (n=4000).

### Detecting Lyme Disease Tweets Using Transformer-Based Classifiers

The training and validation data sets were used to fine-tune a set of pretrained transformer-based classifiers so that they could identify whether a new unknown tweet is a potential Lyme disease case. Recently, transformer-based models have been highly efficient in various NLP applications. Specifically, the BERT model [74], which was developed by Google AI Language in 2018, was an advancement in the transformer paradigm as it allows for the learning of token representations in both left-to-right and right-to-left directions. BERT pretraining incorporates a masked language model and next-sentence prediction, with the ability to adjust or fine-tune its parameters on other relevant data sets. 

Most BERT classifier variants were typically trained to understand tweet semantic content and context to generate word embedding representations. They are language models that require a sequence of tokens as input. Thus, the cleaned tweets were fed into word-piece tokenizers, which converted them into a sequence of lemmatized tokens peppered with 3 special tokens: [CLS], which stood for classification and was typically the first token of every sequence; [SEP], which described to the pretrained language model which token belongs to which sequence; and [PAD], which was used to fill the unused token slots to ensure that the maximum token length was met [75]. When a token sequence exceeded the maximum length, it was truncated. Several variants of BERT classifiers have been proposed, but only the 3 most efficient ones were considered in this study: ALBERT, DistilBERT, and BERTweet [76], a light variant of the BERT architecture that enhances training efficiency by factorizing embedding and sharing cross-layer parameters. We used the Albert-xlarge-v2 model, which has 12 repeated layers (called transformer blocks), 4096 hidden dimensions, a 128 embedding size, and 64 attention heads with 235 million trainable parameters. The AlBertTokenizer, which is associated with ALBERT, was used to tokenize each tweet into a sequence of tokens. These tokens were then synchronously fed into ALBERT’s layers, where each layer used self-attention and transmitted its intermediate encoding via a feed-forward network before passing it on to the next transformer encoder block. For each token, the ALBERT model generated an embedding vector. DistilBERT [77] is a small and computationally efficient form of BERT. It is 60% faster than the BERT_base_ model but 40% smaller owing to knowledge distillation during pretraining, all while achieving 97% of its language understanding efficiency. Compared with BERT, the number of layers in its student architecture has been trimmed in half, and token-type embeddings have been eliminated. We used the DistilBERT-base-uncased model, which has 6 layers, 768 hidden nodes, and 66 million unique parameters in total. Furthermore, because DistilBERT does not require token type IDs, it is not necessary to specify which token belongs to which segment. To tokenize the input sentences of the tweets into token sequences, we used the DistilBertTokenizer equipped with the model. The DistilBERT model then outputs an embedding vector for each token. Finally, BERTweet [78] is a recent large-scale artificial intelligence model specifically for English tweets based on BERT. BERTweet was trained on an 80 GB uncompressed corpus containing 850 million tweets streamed from January 2012 to August 2019, and 5 million tweets related to the COVID-19 pandemic, with each tweet containing at least 10 and no more than 64 word tokens. We specifically used the BERTweet-base model, which has 12 layers (transformer blocks) with a hidden size of 768 and a total of 110 million unique parameters. The model’s creators produced BertweetTokenizer, which was used to tokenize the tweets’ input texts into sequences of tokens. The BERTweet model also generates an embedding vector for each token. On holy-grail NLP tasks, such as entity resolution and short text classification, BERTweet outperformed state-of-the-art baselines, such as RoBERTa_base_ and XLM-R_base_ [78].

### Embedding Enhancement

Emojis are used to express emotions succinctly and are popular communication tools on social media. Several potential Lyme disease patients frequently self-report their symptoms in tweets that combine word and emoji sequences. Thus, excluding emojis during preprocessing could lead to the loss of important information. As a result, we aimed to improve the tweet’s contextual encoding by including its emoji expressions. Traditionally, the more efficient way of leveraging emojis to enrich the feature embedding of a tweet is to use any emoji package, such as demoji, to convert emoji icons into sentiment words. The corresponding sentiment words are then substituted for emoji icons inside the tweets, resulting in tweets consisting of only word sequences that can be fed as input to the tokenizers associated with the BERT-based models.

### Ethical Considerations

The use of tweets for academic research purposes is provided for in Twitter’s development policy and the consent form signed by users [61]. Social media data are publicly accessible data. However, in accordance with Twitter’s terms of use and to protect users’ privacy, all personal information and tweets were deleted. There is no path or link from this paper (or any supplementary material related to this paper) to any individual tweets, users, or IDs.

## Results

We initially compared the accuracy of the NLP classifier models (ALBERT, BERTweet, and DistilBERT) for detecting potential Lyme and non-Lyme disease tweets with the following state-of-the-art classification models: AdaBoost [79], random forest (RF) [80], logistic regression (LR) [81], Multilayer Perceptron Neural Network (MLP) [82], support vector machine (SVM) [83], k-nearest neighbors (KNN) [84], Quadratic Discriminant Analysis (QDA) [85], and Naive Bayes (NB) [82]. Using the term frequency-inverse document frequency (TF-IDF) vectorization method [86], the tweet embeddings were generated and then fed into the classifiers, except for the 3 transformer-based classifiers associated with their tokenizers. As reported previously [71], we regularized our classifier models to avoid overfitting by including extreme penalizing terms in the objective functions with L1/L2 together with solvers like liblinear, lbfgs, and saga [62,64]. The learning rate was 0.01, and the number of estimators was 100. Since Twitter data are short text data, we chose the Adam algorithm, which has been shown to better handle potential problems associated with such data and has low sensitivity to the learning rate [74,87]. In order to maximize the likelihood estimation, we also evaluated the loss function by implementing binary cross-entropy [71]. As mentioned previously [71], we used a learning rate of −2 × 10^−5^, a weight decay of 0.001, and a batch size of 64.

To ensure consistent results across evaluations, all the classification models were built using the same training, validation, and test data sets. Specifically, after combining the training and validation data sets, we used 10-fold cross-validation to train the underlying classification models. Thus, 9 of the 10 folds were used in the training phase to iteratively learn the model parameters, and the remaining fold was used for validation. We used all learned classifiers to predict tweet labels during the testing phase and then recorded their confusion matrices on the testing data set to capture the following quantities: (1) the proportion of actual Lyme disease tweets correctly classified as potential Lyme disease cases (ie, true positives); (2) the proportion of actual non-Lyme tweets correctly classified as unrelated to Lyme disease (ie, true negatives); (3) the proportion of actual non-Lyme disease tweets incorrectly classified as belonging to the potential Lyme disease class (ie, false positives); and (4) the proportion of actual potential Lyme disease tweets misclassified as non-Lyme disease tweets (ie, false negatives). We computed several evaluation metrics based on confusion matrices to assess the accuracy of all tested classifiers as follows: classification accuracy [88], which measures the proportion of correct predictions (true positives and true negatives) among all examined tweets; average F1-score [89], which quantifies the likelihood of correctly identifying Lyme-disease tweets; and precision and recall, which quantify the proportion of correctly identified tweets that are actual potential Lyme disease cases and vice versa, respectively. The LR classification was considered to serve as an effective baseline for comparison.

As shown in Table 1, the BERTweet model was the best among all the NLP models included in our study. This model had the highest classification accuracy of 90.0%, average F1-score of 89.3%, precision of 97.1%, and recall of 82.6%. DistilBERT was close to BERTweet and was slightly more accurate than ALBERT. LR performed adequately in classifying tweets about Lyme disease but was significantly less accurate than ALBERT, with a classification accuracy of 76.6% and an F1-score of 76.7%. The classification accuracy scores of the QDA, RF, and AdaBoost models were comparable to those of the LR model, with both RF and AdaBoost having slightly more false negatives and fewer false positives than LR. A false negative was identified with a recall score as low as 62.7%, while a false positive was identified with a precision score as high as 96.5%. AdaBoost was slightly ahead of MLP but comparable to RF, as both AdaBoost and RF had a classification accuracy of 76.2% and an F1-score of 76%. SVM and the baseline LR performed similarly, with roughly the same scores.

**Table 1:** Classification accuracy, average F1-score, precision, and recall for all classification models on the test data set.

Model	Accuracy (%)	F1-score (%)	Precision (%)	Recall (%)
TF-IDF^a^ and AdaBoost	76.6	76.0	96.5	62.7
TF-IDF and random forest	76.6	76.0	96.5	62.7
TF-IDF and logistic regression	76.6	76.7	93.4	65.0
TF-IDF and Multilayer Perceptron Neural Network	76.5	75.9	96.9	62.3^b^
TF-IDF and support vector machine	76.5	76.5	93.4	64.8
TF-IDF and k-nearest neighbors	71.8^b^	79.6	69.4^b^	93.2^c^
TF-IDF and Quadratic Discriminant Analysis	76.6	76.6	93.5	64.8
TF-IDF and Naive Bayes	73.7	75.6^b^	83.7	68.9
DistilBERT^d^	89.2	88.2	96.8	81.0
ALBERT^e^	88.4	87.3	96.6	79.7
BERTweet^f^	90.0^c^	89.3^c^	97.1^c^	82.6

^a^TF-IDF: term frequency-inverse document frequency.

^b^Lowest score value.

^c^Highest score value.

^d^DistilBERT: distilled version of Bidirectional Encoder Representations from Transformers.

^e^ALBERT: A Lite Bidirectional Encoder Representations from Transformers.

^f^BERTweet: Bidirectional Encoder Representations from Transformers for English Tweets.

Notably, KNN had the lowest precision score of 69.4%, producing significantly more false positives than any of the other classifiers tested. However, it also had the highest recall score of 93.2%, providing significantly fewer false negatives. When compared with the QDA, SVM, LR, and AdaBoost models, the NB classifier recall score of 68.9% showed slightly fewer true negatives, and its precision score of 83.7% demonstrated significantly more false positives. Overall, and apart from the transformer-based classifiers, QDA had the most consistent performance when all metrics were considered at once, with a classification accuracy of 76.5%, F1-score of 76.5%, precision of 93.4%, and recall of 64.8%.

Second, we investigated whether the inclusion of emojis improves the contextual encoding and classification of tweets. As described in the Methods section, we first used the demoji library to extract emoji icons and convert them into words to enrich the tweet embeddings. We then repeated the previous procedure to classify the tweets. Overall, BERTweet still outperformed the other tested variants of the BERT classification model, with the highest classification accuracy of 95.2%, average F1-score of 94.9%, precision of 98.8%, and recall of 91.2%. DistilBERT followed BERTweet and was slightly more accurate than ALBERT. The recall score for BERTweet with emojis was 8% higher than its recall score without emojis, and DistilBERT and ALBERT with emojis had recall scores that were at least 9% higher than their recall scores without emojis. The 3 classifiers were also able to reduce the produced false positives by at least 5% when emojis were used. As a result, DistilBERT had a significantly higher F1-score of 93.8% and accuracy of 94.1%, while ALBERT had a higher F1-score of 93% and accuracy of 93.9%. These results are summarized in Table 2.

**Table 2:** Classification accuracy, average F1-score, precision, and recall for the transformer-based classification models on the test data set after including emojis.

Model	Accuracy (%)	F1-score (%)	Precision (%)	Recall (%)
BERTweet^a^	95.2	94.9	98.8	91.2
ALBERT^b^	93.9	93.1	97.3	89.2
DistilBERT^c^	94.1	93.8	97.5	90.4

^a^BERTweet: Bidirectional Encoder Representations from Transformers for English Tweets.

^b^ALBERT: A Lite Bidirectional Encoder Representations from Transformers.

^c^DistilBERT: distilled version of Bidirectional Encoder Representations from Transformers.

Finally, we explored the collected tweets to determine if we could identify certain patterns. After geolocating the tweets, we found that they originated from 46 countries all over the world. The United States, the United Kingdom, Canada, and Australia had the highest number of potential Lyme disease–related tweets and non-Lyme disease tweets, accounting for 97.1% (19,418/20,000) of the total. Remarkably, there were observed spikes in both Lyme disease and non-Lyme disease tweet counts for the United States, as the United States is a hotspot country for Lyme disease. Overall, the greatest proportion of potential Lyme disease–related tweets were from the United States (9827/20,000, 49.1%), whereas 0.2% (43/20,000) were reported from Canada, 0.03% (6/20,000) from Mexico, and 0.01% (2/20,000) from some Caribbean countries, such as Haiti and Jamaica. A total of 0.03% (6/20,000) of the potential Lyme disease–related tweets were reported from some South American countries, including Argentina and Venezuela. Potential Lyme disease cases reported in Europe were from Belgium, Denmark, Estonia, France, Ireland, Luxembourg, Norway, Poland, Sweden, and Switzerland, and represented 0.7% (143/20,000) of all tweets, while 0.3% (55/20,000) were from the United Kingdom. Potential Lyme disease cases reported in Asia were from Indonesia, Iran, the Philippines, South Korea, Taiwan, Thailand, and Vietnam, and represented 0.08% (15/20,000) of the data set. Potential Lyme disease cases from Africa represented 0.005% (1/20,000) of the data set and came from a single country, Sudan. Finally, New Zealand and Australia had 0.09% (18/20,000) of the total potential Lyme disease cases on Twitter, each accounting for 0.02% (4/20,000) and 0.07% (14/20,000), respectively.

Table 3 presents the number of medical symptoms of Lyme disease reported in tweets. Rash, fatigue, tick bite, fever, and arthritis were the most commonly reported symptoms. In contrast, symptoms, such as neck stiffness, numbness, and lymph nodes, were rarely reported. The classified data are available in the GitHub repository of this study [90].

**Table 3:** Top medical symptoms of Lyme disease reported in tweets.

Medical symptoms	Lyme disease–related tweet count
Rash	167
Fatigue	165
Tick bite	130
Fever	109
Arthritis	103
Sleepy	78
Migraine	48
Depression	48
Headaches	47
Carditis	25
Joint pain	20
Memory loss	11
Erythema migrans	8
Nausea	6
Nerve pain	6
Dizziness	5
Vomiting	4
Tingling	3
Palpitation	3
Chills	3
Numbness	3
Lymph nodes	3
Irregular heartbeat	2
Neck stiffness	1
Borrelial lymphocytoma	1

## Discussion

### Principal Findings

The empirical results of this study highlight the improved performance of transformer-based classifiers (ie, BERTweet, DistilBERT, and ALBERT), which we attribute to the following reasons. First, the tweet word embeddings produced by their associated tokenizers were more accurate than those generated by context-independent embedding techniques such as TF-IDF. This is because transformer-based classifiers are based on language models and can better understand the semantic content of short texts such as tweets in different contexts. Unlike TF-IDF, the tokenizers also consider the position and order of words in tweets, which improves their ability to understand the different meanings of individual words. Second, unlike AdaBoost, KNN, and RF, transformer-based classifiers are less affected by noise and redundant words in tweets. Third, transformer-based classifiers can learn nonlinear relationships and complex patterns in tweets because their neural network architecture does not assume linearity between dependent and independent features (unlike LR and SVM), and nonlinearity between the features is often the case in extracted tweets. Finally, transformer-based classifiers differ from both MLP and KNN because they can efficiently handle feature scaling and frequently converge to the global optimum rather than getting stuck in the local minima. This is due to the optimization of the cross-entropy loss function, which is often convex for most weights.

The results also showed that emojis are effective enrichment features to improve the accuracy of tweet embedding. The performance of transformer-based classifiers can be further improved by considering the sentimental semantics of emojis. Since the texts of non-Lyme disease and Lyme disease–related tweets can be similar in some cases, emojis can play important roles in better identifying Lyme disease–related tweets. When emojis are removed during the text cleaning process, some potential Lyme disease–related tweets could be misidentified as non-Lyme ones, resulting in more false negatives. This implies that the inclusion of sentimental or emotional words representing sadness, empathy, and encouragement emojis could significantly assist transformer-based classifiers in distinguishing potential Lyme disease–related tweets from non-Lyme disease tweets.

The classification of the 20,000 tweets used in this study showed a high volume of potential Lyme disease–related tweets in the United States, the United Kingdom, and Canada. This may be due to 2 reasons. First, Lyme disease is spreading, and second, a focus solely on English tweets may limit the collection of tweets from non-English speaking countries. Furthermore, in the case of symptoms, borrelial lymphocytoma, palpitations, tingling, nausea, and neck stiffness are rarely reported. In fact, there are differences in the clinical manifestations seen in North America and in European countries. For example, Lyme arthritis and carditis are mainly found in North America, while borrelial lymphocytoma and neurological symptoms (neck stiffness, numbness, etc) are found in European countries. Erythema migrans, which is the most common clinical symptom, is not among the most common symptoms reported on Twitter because the general population tends to refer to this symptom as a rash. These findings correlate with the geographic distribution of the clinical manifestations of Lyme disease throughout the literature [46,47,91].

Lyme disease is endemic in both the United States and Europe. Although CDC surveillance has reported over 30,000 cases annually, other studies have estimated 476,000 cases yearly in the United States [10,47,48]. In Europe, over 200,000 cases of Lyme disease are being reported yearly [92,93]. The results of our study are in line with the literature since Lyme disease–related tweets originate mainly from the United States. However, it is difficult to compare these results, given that only English-language tweets were used in this study and that the distribution of Twitter users is related to geographical location. Our study is the first to provide a pretrained, organized, and labeled Lyme disease–related data set with an emoji component, which can be used to quantify and compare the performance of different methodological approaches in future Lyme disease–related work. This will allow for consistency in future research and improve digital surveillance of Lyme disease. For example, a sudden increase in Lyme disease–related activity on Twitter or other social media platforms may indicate the beginning of an increase in cases, which could justify the promotion of tick bite prevention measures in the indicated geographical area. For example, we found studies reporting erythema migrans in combination with *Borrelia burgdorferi* sensu lato antibodies after tick bite in some patients from regions in the Caribbean, who do not have any travel history to Lyme disease–endemic regions [94,95]. Despite the controversy surrounding the presence of Lyme disease vectors in the Caribbean [94], these findings agree with our study results that showed some Lyme disease–related tweets in the region, suggesting a need for further investigation on the presence of Lyme disease vectors in the Caribbean. Finally, to be able to compare our results to other early warning systems, we extracted Lyme disease reports on ProMED (which is the largest early warning system for emerging diseases in the world). A comparison of the Lyme disease cases identified in these reports with our data revealed some similar results. In fact, the majority of ProMED Lyme disease reports came from the United States (57%); Canada (25%); the United Kingdom (7%); and some European countries, such as Finland, Belgium, and Austria (9%). These results are in line with our findings, with some disparities in terms of ranking. Indeed, in our results, the United Kingdom ranks second for potential Lyme disease cases, while Canada ranks third. However, the trend holds for the United States and other European countries.

### Limitations

Our study has several limitations. First, the collection of Twitter data through the Twitter API is suggested to have a selection bias, since only 1% of the data are accessible. Furthermore, because the data collected are randomly generated, the data may not reflect the reality of Lyme disease conversations on twitter. This study aimed to limit this bias by collecting data over a long period of time. Additionally, social media data are highly susceptible to media coverage, so tweets about Lyme disease may be driven by media coverage rather than disease incidence.

While we provided a pretrained Lyme disease–related tweet data set, our results only reflected Lyme disease–related English tweets. As we excluded tweets published in languages other than English from our data set, we were not able to access all potentially relevant Twitter discussions about Lyme disease from non-English speaking countries where Lyme disease is endemic.

Although emojis have general meanings, their usage mainly depends on other factors, such as cultural background, linguistic factors, and gender [96]. Since our study only focused on the sentimental semantics of emojis, our model may have erroneously assigned a certain meaning to emojis different from what the tweet author intended. Therefore, our results should be interpreted with some caution. However, since our model was trained with labeled tweets, we believe that the mislabeling of some emojis did not significantly affect the performance of our model.

While we were able to geolocate the tweets, users may not register with their exact location or may register with a wrong location due to safety concerns [97]. To reduce such bias, we did not map granular tweet-specific locations, but rather expanded the spatial distribution to the country level to reduce the risk of location errors.

One common limitation of using social media data is that tweeting does not necessarily equate to the occurrence of Lyme disease [63,98,99]. Thus, our model may have included tweets about Lyme disease but not actual cases of Lyme disease. However, we believe that the model has been well-trained with various keywords related to Lyme disease, therefore improving the performance of BERT transformer models.

Additionally, according to Marques and other collaborators, *Ixodes* ticks can carry two or more pathogens and are capable of transmitting them in a single bite, thereby resulting in co-infection [4,10,100]. Given the increased public awareness of Lyme disease compared to that of other tick-borne diseases, the public use of Lyme disease as an umbrella term to describe any tick-borne disease may confound the results of this study. Specifically, Lyme disease–related Twitter discussions in endemic tick-borne disease regions may not actually be solely associated with potential Lyme disease cases when there is a risk of infection by non-Lyme disease–related pathogens [26]. Therefore, when interpreting the results of our study, these limitations should be considered.

### Conclusions

The early detection of potential Lyme disease cases is essential to limit its increase and improve the efficiency of medical care. Given the growing importance of social media as a source of information about infected cases, platforms, such as Twitter, can provide simultaneous updates on the Lyme disease epidemic. This makes the use of such novel data for Lyme disease prediction and surveillance an important but underexplored challenge in the field of health informatics. In this work, we propose a Lyme disease detection system that is primarily a transformer-based classifier that uses data from self-reported tweets to identify potential cases of Lyme disease. While Twitter data were the focus of this work, the proposed system can be easily adapted to other social media text–based platforms like Reddit. We suggest that future research should focus on collecting social media data from both English and non-English texts to improve the knowledge of potential Lyme disease cases, as some of the countries with the highest incidence of Lyme disease are non-English speaking countries. Additionally, as our model was able to identify some Lyme disease–related tweets from regions that typically have a low reported incidence of Lyme disease (ie, African countries and the Caribbean), we believe that the results are valuable for informing emerging Lyme disease surveillance activities in these geographical areas. Despite these limitations, our study provides a steady performance model with publicly available data for researchers and policymakers to identify trends in Lyme disease discussions on social media.

## Abbreviations

ALBERT: A Lite Bidirectional Encoder Representations from Transformers
API: application programming interface
BERT: Bidirectional Encoder Representations from Transformers
BERTweet: Bidirectional Encoder Representations from Transformers for English Tweets
CDC: Centers for Disease Control and Prevention
DistilBERT: distilled version of Bidirectional Encoder Representations from Transformers
KNN: k-nearest neighbors
LR: logistic regression
MLP: Multilayer Perceptron Neural Network
NB: Naive Bayes
NLP: natural language processing
QDA: Quadratic Discriminant Analysis
RF: random forest
SVM: support vector machine
TF-IDF: term frequency-inverse document frequency

## Data Availability

The data sets analyzed in our study are available on GitHub [90]. However, to protect and ensure Twitter users’ privacy, we only share the location and labels.
